# Brown patches and erosions with an erythematous base

**DOI:** 10.1016/j.jdcr.2025.05.021

**Published:** 2025-06-18

**Authors:** Madisen A. Swallow, Rebecca Fine, Alana Deutsch, Jeffrey Gehlhausen, Noel Turner, Shawn E. Cowper, Sarika M. Ramachandran, Caroline A. Nelson, Mary M. Tomayko

**Affiliations:** a Yale School of Medicine, New Haven, Connecticut; b Department of Dermatology, Yale School of Medicine, New Haven, Connecticut; c Department of Pathology, Yale School of Medicine, New Haven, Connecticut

**Keywords:** cutaneous-only pemphigus vulgaris, pemphigus vulgaris

A 78-year-old man presented with a blistering rash consisting of numerous red to brown patches and erosions with an erythematous base and secondary crusting (Fig 1). Notably, the ocular, nasal, oropharyngeal, and anogenital mucosae were clear. Intact vesicles extended and ruptured when touched. Antidesmoglein 1 and antidesmoglein 3 antibodies were detected at elevated levels of 117 U/mL and 96 U/mL, respectively. Histopathology demonstrated focal acantholysis with mild spongiosis, an overlying scale crust, and a dermal inflammatory infiltrate composed of lymphocytes and a few eosinophils (Fig 2). Direct immunofluorescence revealed netlike and focal punctate immunoglobulin G (IgG) deposition (Fig 3) and C3 positivity.

**Fig 1:**
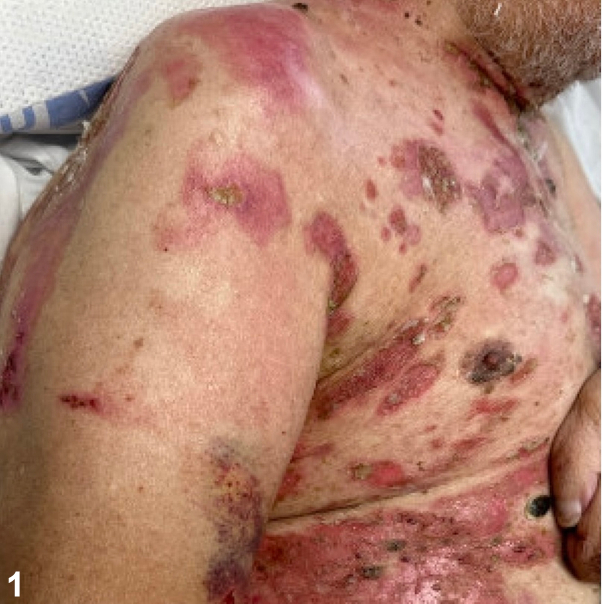

**Fig 2:**
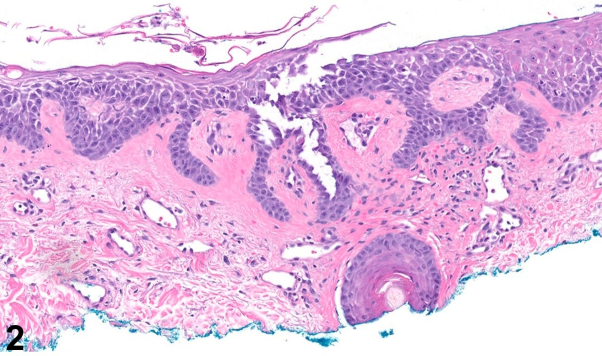

**Fig 3:**
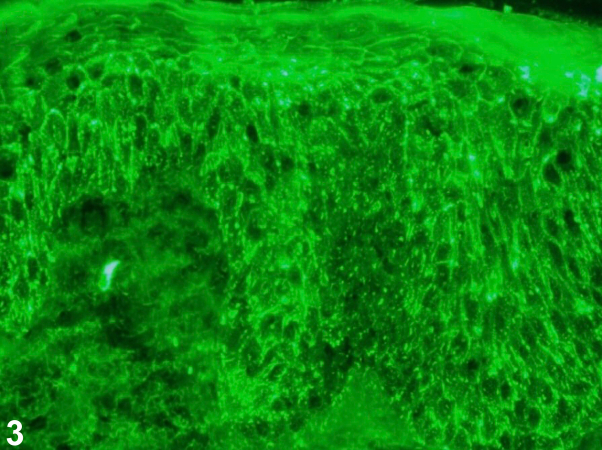

**Question 1: What is the most likely diagnosis?**

A. Bullous pemphigoid
B. Stevens-Johnson syndrome/toxic epidermal necrolysis (SJS/TEN)
C. Staphylococcal scalded skin syndrome (SSSS)
D. Pemphigus vulgaris (cutaneous-only subtype)
E. Pemphigus foliaceus

**Answers:**

A. Bullous pemphigoid – Incorrect. Although bullous pemphigoid is also an autoimmune blistering disorder, patients with bullous pemphigoid typically have a negative Nikolsky sign and usually have autoantibodies to BP180 and BP230.
B. Stevens-Johnson syndrome/toxic epidermal necrolysis (SJS/TEN) – Incorrect. Despite a positive Nikolsky sign seen in this patient, the patient lacked any mucosal involvement at presentation, which would be very uncommon for SJS/TEN. Furthermore, patients with SJS/TEN do not create autoantibodies to desmoglein.
C. Staphylococcal scalded skin syndrome (SSSS) – Incorrect. Although SSSS also presents with a positive Nikolsky sign, blistering lesions, and mucosal site sparing, SSSS is caused by exfoliative toxins that inhibit desmoglein 1 functioning rather than autoantibodies to this protein as seen in pemphigus.
D. Pemphigus vulgaris (cutaneous-only subtype) – Correct. Cutaneous-only pemphigus vulgaris presents with more superficial, pemphigus foliaceus-like crusted erosions, histology most often demonstrates suprabasilar acantholysis, and serology exhibits both antidesmoglein 1 and antidesmoglein 3 IgG autoantibodies.^1^
E. Pemphigus foliaceus – Incorrect. Pemphigus foliaceus usually presents with superficial erosions with scaling and crust, but only antidesmoglein 1 antibody positivity.

**Question 2: Approximately how common is cutaneous-only pemphigus vulgaris?**

A. 5% to 20% of pemphigus vulgaris cases
B. 25% to 50% of pemphigus vulgaris cases
C. 60% to 75% of pemphigus vulgaris cases
D. 75% to 85% of pemphigus vulgaris cases
E. 95% to 100% of pemphigus vulgaris cases

**Answers:**

A. 5% to 20% of pemphigus vulgaris cases – Incorrect. This percentage is too low.
B. 25% to 50% of pemphigus vulgaris cases – Correct. A 2015 study of the International Pemphigus and Pemphigoid Foundation registry demonstrated that at the time of study participation, 24.5% of pemphigus vulgaris patients reported cutaneous-only lesions.^2^ Furthermore, cutaneous-only pemphigus vulgaris has been described as a transient phenotype that emerges from, or progresses into, other clinical presentations of pemphigus.^1^ This transient presentation was also previously demonstrated by Sielski et al who reported that when evaluating 159 pemphigus vulgaris patients, 84 had cutaneous-only lesions, of whom only 7 reported no history of mucosal lesions.^3^ Interestingly, our patient developed a new mucosal erosion on the tip of tongue 6 weeks after the rash first started and following treatment initiation, emphasizing the importance of ongoing surveillance for mucosal involvement in any patient with pemphigus vulgaris presenting with cutaneous-only lesions.
C. 60% to 75% of pemphigus vulgaris cases – Incorrect. This percentage is too high.
D. 75% to 85% of pemphigus vulgaris cases – Incorrect. This percentage is too high.
E. 95% to 100% of pemphigus vulgaris cases – Incorrect. This percentage is too high.

**Question 3: How can the desmoglein compensation theory explain why cutaneous-only pemphigus vulgaris patients do not have mucosal lesions?**

A. Although patients produce antibodies to desmoglein 1, desmoglein 1 expression is lesser in the skin than in oral mucosa
B. Although patients produce antibodies to desmoglein 3, desmoglein 3 expression is lesser in the skin than in oral mucosa and patients can produce antibodies with varying pathogenic potential
C. Patients produce antibodies only to desmoglein 1, but not to desmoglein 3
D. Patients produce antibodies only to desmoglein 3, but not to desmoglein 1
E. The manifestations of cutaneous-only pemphigus vulgaris cannot be explained by the desmoglein compensation theory

**Answers:**

A. Although patients produce antibodies to desmoglein 1, desmoglein 1 expression is lesser in the skin than in oral mucosa – Incorrect. Desmoglein 3 expression is lesser in the skin rather than desmoglein 1.^1^^,^^4^
B. Although patients produce antibodies to desmoglein 3, desmoglein 3 expression is lesser in the skin than in oral mucosa and patients can produce antibodies with varying pathogenic potential – Correct. Studies have shown that desmoglein 3 expression is lesser in the skin than in oral mucosa.^1^^,^^4^ Cutaneous-only pemphigus vulgaris patients produce antibodies with weak pathogenic potential that block functioning in the skin but cannot inhibit functioning in the mucosa.^1^ Antidesmoglein 3 IgG monoclonal antibodies from mouse models have demonstrated varying pathogenic activity, with only some antibodies inducing blisters.^1^^,^^5^
C. Patients produce antibodies only to desmoglein 1, but not to desmoglein 3 – Incorrect. The production of antibodies to only desmoglein 1 is the pathology underlying pemphigus foliaceus and explains why these patients present with cutaneous superficial erosions with scaling and crust with mucosal sparing.
D. Patients produce antibodies only to desmoglein 3, but not to desmoglein 1 – Incorrect. The production of antibodies to only desmoglein 3 is the pathology underlying the mucosal-dominant subtype of pemphigus vulgaris and explains why the classic presentation for these patients includes mucosal blisters and erosions.
E. The manifestations of cutaneous-only pemphigus vulgaris cannot be explained by the desmoglein compensation theory – Incorrect. These patients create antibodies with a range of pathogenicity and there is less expression of desmoglein 3 in the skin.

## Conflicts of interest

None disclosed.
